# Chemotaxis Assay for *Marsupenaeus japonicas* Hemocytes and Application for the Development of an Oral Immunostimulant Against White Spot Syndrome Virus

**DOI:** 10.3389/fcell.2020.00046

**Published:** 2020-02-07

**Authors:** Takashi Imai, Yukinori Takahashi

**Affiliations:** ^1^Department of Infectious Diseases and Host Defense, Graduate School of Medicine, Gunma University, Maebashi, Japan; ^2^Department of Applied Aquabiology, National Fisheries University, Shimonoseki, Japan

**Keywords:** chemotaxis, Boyden chambers, hemocytes, kuruma prawn/shrimp, white spot syndrome virus, immunostimulant, laminaran, β glucan

## Abstract

The chemotactic activity of vertebrate leukocytes is an important host-defense mechanism. However, chemotaxis of invertebrate immune cells, particularly those of shrimp species, is incompletely understood and critically understudied. In this study, we aimed to optimize the conditions for a Boyden chamber chemotaxis assay using hemocytes (granulocytes) from cultured kuruma shrimp, *Marsupenaeus japonicas* (Mj) and the optimal conditions were: 5 μm-pore-size Polyvinylpyrrolidone membrane; culture buffer at pH 7.0; and chemotactic factor N-formyl-methionyl-leucyl-phenylalanine (fMLP) 10^–8^ mol/L; 4 h incubation time. We then applied the chemotaxis assay to develop an oral immunostimulant against white spot syndrome virus (WSSV), which results in high mortality rates in several shrimp species worldwide. We focused on the kelp *Laminaria japonica*, as this species contains immunostimulative molecules such as β-glucan. We prepared Heat Extracts (HE) and Crude Laminarans (CL) from kelp using hot water and hydrochloric acid extraction methods, respectively. HE and CL ware mixed with normal shrimp feed. Kelp extracts were orally administered for 7 days, and hematocyte chemotaxis toward fMLP was compared. No difference was detected between control and kelp extracts on day 3, but HE stimulated chemotaxis 2-fold and CL stimulated chemotaxis 3-fold relative to control on day 7 after initiating administration. Kelp extract administration protected against WSSV exposure. Finally, we identified that Kelp extracts stimulated hematocyte superoxide production on days 3 and 7, and increased hematocyte phagocytosis and phenol oxidase activity on day 7 after administration. We concluded that the chemotaxis assay is informative in assessment of shrimp hemocyte immunological activity, and is applicable to the development of immunostimulants against shrimp infectious diseases.

## Introduction

Vertebrate and invertebrate animals have host defense systems, including cellular and humoral factors, to protect the body (Loker et al., 2004; Wong, 2015). The cellular response is important protection against infectious diseases and invasive foreign substances. The invertebrate immune system differs from that of vertebrate animals in that invertebrates do not have acquired immunity, such as T cells and B cells, but invertebrates do have hemocyte in the hemolymph for innate immunity. Invertebrate hemocytes are multifunctional, and are capable of chemotaxis/migration, phagocytosis, encapsulation/nodulation, melanization by phenol oxidase activation, production of reactive oxygen species, and production of antimicrobial peptides (Loker et al., 2004).

Chemotaxis is a fundamental function of the cell, and can be measured by agar-plate assays (Luo et al., 2008) and two-chamber assays. Chemotaxis is induced by chemokines or chemoattractants, which bind to chemokine receptors and trigger intracellular signaling to induce cell migration (Kurata et al., 2014; Alsharif et al., 2015). Two-chamber assays may use Boyden chambers, Zigmond chambers (Zigmond, 1988), Dunn chambers (Dunn et al., 1978), Multi-well chambers (Thomsen and Jensen, 1991), and Capillary techniques (Webb et al., 2018). The Stepen Boyden chamber was developed by Dr. Boyden in 1962 (Boyden, 1962; Falasca et al., 2011), and is widely used for chemotaxis assays. The Boyden chamber has two compartments separated by a membrane filter. To evaluate the cell migration due to chemotaxis, the test solution is added to one compartment and the cells are added to the other compartment.

Very few papers have described chemotaxis of invertebrate hemocytes (Garcia-Garcia et al., 2009; Moreira et al., 2011; Evans and Wood, 2014), particularly those of marine invertebrates (Fawcett and Tripp, 1994; Yip et al., 2001; Pinto et al., 2003). We are not able to apply the conditions of vertebrate chemotoxis such as mouse directly to marine invertebrate, for example, because it is necessary to consider the difference between Warm-Blooded and Cold-Blooded animals. Further, no prior studies have optimized assay conditions to measure shrimp hemocyte chemotaxis. Yip et al. reported that shrimp hemocytes derived from *Penaeus penicillatus* exhibited chemotaxis toward chemoattractant but did not report assay optimization in detail (Yip et al., 2001).

It is necessary for immune cells in all animals to recognize invasion of foreign bodies and initiate host defenses, the migratory ability of hemocytes (granulocytes) in shrimp is an important function for host defense. In this report, we tested different conditions and optimized the chemotaxis assay using hemocytes (granulocytes) isolated from kuruma prawn/shrimp *Marsupenaeus japonicas* (Mj).

We then examined whether we could apply this assay for development of an oral immunostimulant against shrimp infectious disease. White spot syndrome (WSS) is a viral disease with a high mortality rate, and is a major challenge for shrimp production worldwide. In Japan, infected juvenile Mj were imported from China in 1993, and killed 6.3 million cultured Mj in Japan. The following year, WSS expanded into all of Japan, and Mj production decreased by 50%, from 3020 to 1519 tons (Nakano et al., 1994). The WSSV agent is 111–152 nm in diameter, 375–404 nm in length, and is a rod-shaped, enveloped double-stranded DNA virus of 293 kbps. WSSV is classified into the family *Nimaviridae* and Genus *whisporirus* (Li et al., 2019).

No shrimp anti-viral medications are currently commercially available, necessitating alternative approaches for treatment and prevention of viral infection. To prevent WSSV, disinfection with chlorine, iodine egg disinfection, diagnosis of viral infection by PCR and elimination of wild crustaceans in culture ponds has been utilized in seeding plants and aquaculture fields (Takahashi et al., 1994). These prophylactic measures have decreased damage to shrimp production, but WSSV and other viral infections remain problematic to shrimp aquaculture worldwide. Because shrimp lack memory cells such as T and B cells, vaccine strategies are not suitable to induce antigen-specific immune responses in shrimp. On the other hand, antigen non-specific activation of innate immune cells such as hemocytes and granulocytes with immunostimulants could be useful in activating the host defense system against infectious disease.

In the present study, we extracted β-glucan (Laminarans) from the kelp species *Laminaria japonica*, and used a chemotaxis assay to examine activation of hemocytes from Mj fed with kelp extracts. Our findings were suggestive of the potential utility of kelp extracts as an immunostimulant in shrimp to protect against WSSV.

## Materials and Methods

### Animals

Cultured kuruma prawn/shrimp *Marsupenaeus japonicas:* Mj (average body weight 20 g) were purchased from Matsumoto Fishery (Miyazaki, Japan). Fifteen Mj were housed in a tank (60 × 40 × 30 cm) with 5 cm of sand and equipped with an underwater filtration system, and allowed to adapt for one week prior to beginning the experiment. The daily water conversion rate was set at about 50%, and the water temperature during the experiment was set at 23–24°C. All experiments were reviewed by the Committee for Ethics on Animal Experiments in the National Fisheries University, and carried out under the control of the Guidelines for Animal Experiments in the National Fisheries University, Japanese Law (No. 105) and Notification (No. 6) of the Government.

### Isolation of Hemocyte (Granulocyte)

Approximately 1 ml of hemolymph (correspond to mammalian blood and lymph) was collected from the base of walking leg of Mj using a syringe containing 3 ml of anticoagulant EDTA (27 mM sodium citrate; 336 mM sodium chloride; 115 mM glucose, EDTA ⋅ disodium; pH 7.0). The collected hemolymph was transferred to a plastic test tube and mixed by inverting. Two ml of each sample was gently placed on Percoll (Sigma) continuous density gradients (60% Percoll in 3.2% NaCl and pre-centrifuged at 35,000 × *g* at 5°C for 20 min), then samples on the Percoll were centrifuged at 1,700 × *g* at 5°C for 10 min, and the bottom layer of the Percoll which containing hemocytes (granulocytes) was collected in a silicon-treated glass test tube. Samples were resuspended with 3.2%NaCl and centrifuged at 400 × *g* at 5°C for 10 min, and the precipitate was washed with culture buffer (490 mM sodium chloride, 5 mM magnesium chloride, 8 mM magnesium sulfate, 15 mM calcium chloride, 9 mM potassium chloride, 5.5 mM Glucose, 10 mM HEPES (pH 7.0, Sigma) by centrifuge at 400 × *g* at 5°C for 10 min. The granulocyte concentration was adjusted to 5 × 10^5^ cells/ml using a culture buffer solution.

### Phagocytosis Assay

Granulocytes (200 μL of a 1 × 10^5^ cells/ml suspension) were placed in the cover glass and were incubated for 20 min, and then unattached cells were washed three times with culture buffer. Heat killed yeasts (2 ml) were adjusted to 1 × 10^7^ cells/ml and added to the granulocyte and incubated for 2 h, followed by washing five times with culture buffer and fixed with 20% formalin for 20 min and then stained with Giemsa solution and a sample was observed under the microscope. Three specimens were prepared per Mj, and at least 200 hemocytes were evaluated per one specimen.

### Boyden Chamber Method

**FIGURE 1:**
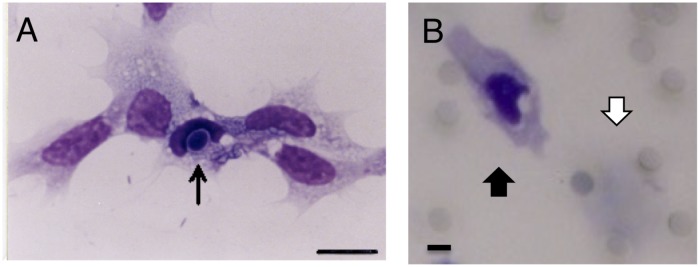
Phagocytosis and chemotaxis of hemocytes (granulocytes) from *Marsupenaeus japonicus*. **(A)** Granulocyte phagocytizing yeasts. Yeast is indicated by the arrow. A Giemsa-stained sample is shown after 2 h incubation with heat-killed yeast on the glass. Scale bar: 10 μm. **(B)** A migrated granulocyte. Chemoattractant was placed on the bottom compartment, and granulocytes were added to the upper compartment and incubated for 4 h at 24°C in the Boyden chamber with PVP membrane. The membrane was fixed with 20% formalin and stained with Giemsa solution. A migrated granulocyte is indicated by a black arrow (lower surface of the membrane), and a pre-migrated granulocyte is indicated by a white arrow (upper surface of the membrane). Scale bar: 5 μm, pore size: 5 μm.

Culture buffer or chemotactic factor was placed in the lower chamber of Boyden Chamber (NEURO PROBE, Blind Well Chamber), and a membrane filter (WHATMAN) was placed between the lower chamber and the upper chamber. Granulocytes (50 μL of a 5 × 10^5^ cells/ml suspension) were placed in the upper chamber, incubated at 24°C for 4 h, in some experiments, fMLP (N-formyl-methionyl-leucyl-phenylalanine, Sigma) which is known as chemoattractant for neutrophils and activator for macrophage (Lindemann et al., 2015) were placed at bottom portion as chemotactic factor. The filter was subsequently removed and transferred to a 24-well cell culture plate with the migrating surface of the granulocytes (the surface facing the lower chamber) facing up. Filters were then fixed with fixation solution (20 ml of formalin and 80 ml of 3.2% sodium chloride solution with 10 g of sucrose) for 20 min, gently washed with culture buffer and dried. Subsequently, 1 ml of Giemsa solution (Merck, diluted 1:50 with PBS) was added, incubated for 5 min, then gently washed with distilled water and dried. The granulocyte migration side of the filter was faced upward and sealed with mounting medium (Eukitt, Cosmo-bio, Japan) dropped on the slide glass. Five filter specimens were prepared per Mj, and the specimen was observed using a microscope (400 times optical magnification, Figure 1B). The migrating hemocytes on the lower surface of filter were counted in random 10 fields of view, and the average value was obtained (mean ± SE). When it needs to see adherent cell, both sides of each filter were counted. All experiments were done at 24°C and repeated at least twice with five shrimps.

### Checkerboard Assay

Using the Boyden chamber method, a checkerboard assay was conducted as described previously (Zigmond and Hirsch, 1973) in order to clarify whether chemotactic factors elicit migration of test cells.

**FIGURE 2:**
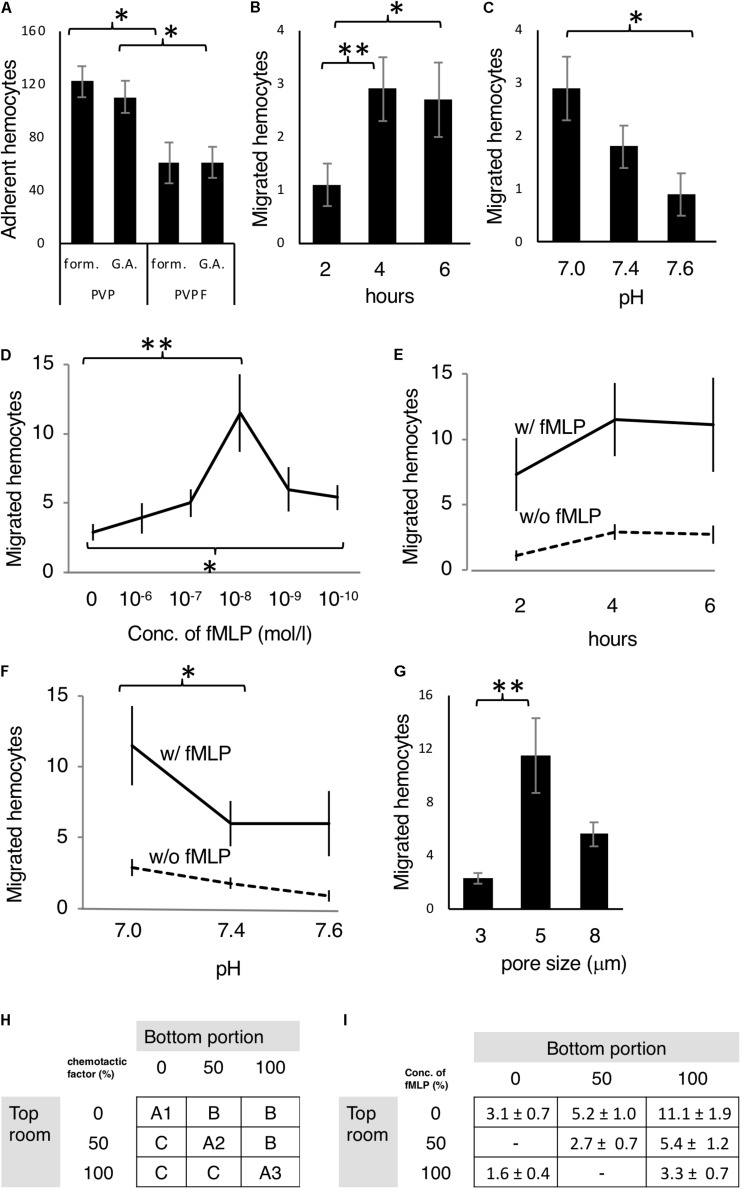
Optimization of the chemotaxis assay. **(A)** Mj hemocytes adhering to both sides of the filter (PVP or PVPF) in the Boyden chamber fixed with formalin (form) or glutaraldehyde (G.A.). **(B,C)** Random migrating hemocytes from the upper compartment through the filter to the lower compartment of the Boyden chamber after **(B)** 2, 4 and 6 h incubation, and **(C)** in culture buffer adjusted to pH 7.0, 7.4 and 7.6. **(D–F)** Optimization of the chemoattractant fMLP. **(D)** Different concentrations fMLP at 0,10^–6^,10^–7^,10^–8^,10^–9^,and 10^–10^ M (4 h, pH 7.0) were assessed. **(E)** Incubation times of 2, 4, and 6 h (fMLP: 10^–8^ M, pH 7.0) were assessed. **(F)** Incubation in culture buffer adjusted to pH 7.0, 7.4, and 7.6 (fMLP: 10^–8^ M, 4 h) was assessed. **(G)** Different pore sizes of the filter, including 3, 5, and 8 μm were examined. **(H)** Checkerboard assay, which was principle of the assay outlined in materials and methods. **(I)** Test with fMLP (10^–8^ M at 100% conc., 4 h, pH 7). All data are expressed as the average (mean ± SD) of total hemocytes counted on the filter surface (**A:** both sides, **B–I:** bottom side) in 10 microscope fields (*N* = 5). ^∗^*P* < 0.05, ^∗∗^*P* < 0.01.

The outline of the checkerboard assay is shown in Figure 2H. A cell suspension in which chemotactic factor was diluted with culture buffer was placed in the upper chamber. Only the chemotactic factor diluent was placed in the lower chamber. As shown in A1 to A3 of Figure 2H, the chemotactic factors in the upper chamber and the lower chamber have the same concentration. A1 is random migration; A2 to A3 shows chemokinensis (activation of cells is stimulated by the substance, but there is no directionality). Also, in B, because the concentration of the chemotactic factor in the lower chamber was higher than that of the upper chamber, the cells that migrated to the lower chamber were measured as cells showing migratory ability. In C, because the concentration of the chemotactic factor is higher in the upper chamber than in the lower chamber, migration of granulocytes was negligible. Establishing the relationship of B > C verified that chemotaxis was occurring.

### Extraction and Administration of Kelp β-Glucan

**FIGURE 3:**
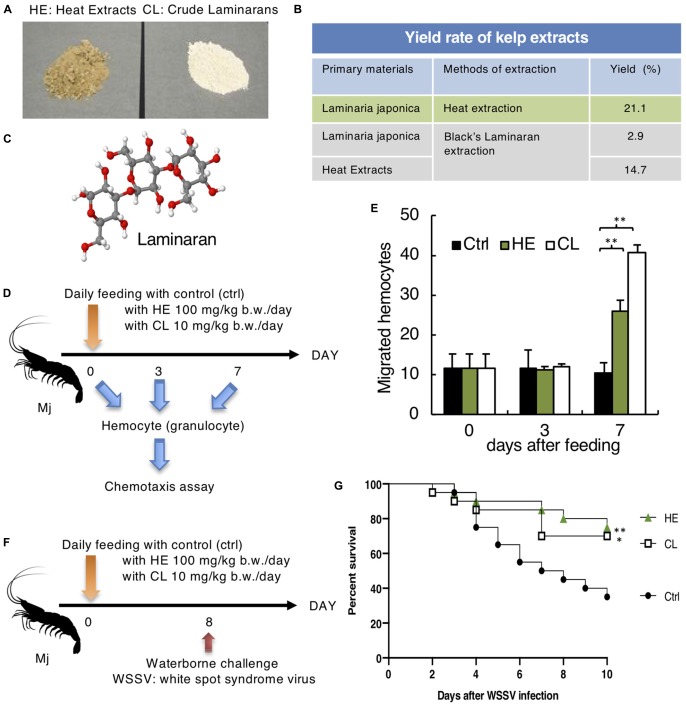
Effect of *Laminaria Japonica* kelp extracts on Mj hemocyte activation and defense against WSSV. **(A)** Heat extracts (HE) and Crude Laminarans (CL) extracted from Kelp are shown. **(B)** Yield rate of kelp extraction protocols. **(C)** Laminaran 3D model (H: white, O: red, C: gray). **(D)** Protocol for kelp extract feeding and chemotaxis assay. **(E)** Effect of kelp extract on chemotaxis toward fMLP after 0, 3 and 7 days of feeding. *N* = 6. ^∗∗^*P* < 0.01. **(F)** Kelp extract was fed every day and Mj were water-bone challenged with WSSV on day 8. **(G)** Survival rates are shown. Data are pooled from two independent experiments with *N* = 20/group. ^∗∗^*P* = 0.01 (ctrl vs. HE), ^∗^*P* = 0.036 (ctrl vs. CL).

Dried *Laminaria japonica* from Hokkaido, Japan was purchased. For the heat extract (HE), after cutting the kelp into approximately 2 mm squares, 20–30 times the weight of water was added, and the mixture was heated at 90–96°C for 2 h. During the heating process, the mixture was agitated to avoid scorching. After heating, the temperature was allowed to cool to room temperature, the mixture was centrifuged (37000 × *g*, 15 min, 4°C), and the supernatant was collected and filtered with a 5 μm filter. Subsequently, the filtrate was placed in an eggplant-type flask pre-cooled at −80°C for 20 min, and the flask was placed in a container containing a mixture of ethanol and dry ice and rotated in order to adhere the filtrate to the inner wall of the flask. The sample-containing flask was attached to a freeze dryer (Eyela Freeze Dryer Fd-80, Tokyo Rika Kikai, Japan), and subjected to freeze drying for 24 h. Thereafter, the dried extract was transferred to a mortar and ground with a pestle to recover the powder (Heat Extracts: HE, Figure 3A left).

Crude laminarin (CL) was purified in accordance with Black’s method (Black, 1965). Briefly, 250 ml 1M hydrochloric acid was added to 26 g dried kelp product or 26 g HE (two different starting materials were used to compare the yields), stirred at 4°C for 2 h, and centrifuged at 40000 × *g*, 4°C for 10 min. The residue was washed with 50 ml 0.05 M hydrochloric acid and centrifuged, and this process was repeated twice. The supernatant and washings were combined, ethanol was added to a concentration of 85%, and the mixture was stored overnight in a cold room. The precipitate was collected by centrifugation, washed twice with ethanol and once with ether, and then air dried. Thereafter, the residue was crushed to obtain CL (Figure 3A, right). Kelp extracts were mixed with normal shrimp feed and orally administrated to Mj. HE was administered at 100 mg/kg/day, and CL was administered at 10 mg/kg/day.

### WSSV Infection

Inoculation of WSSV was based on previous research (Takahashi et al., 2000) with a small modification. In brief, dying shrimps with WSS are stored at −80°C as a source of infection. Three shrimps’ head (cephalothorax) without carapace were crushed with homogenizer in 40 ml of sterilized seawater and centrifuge at 1,700 × *g* at 4°C for 10 minutes. The supernatant was filtrated with 0.45 μm and diluted 16,000 times with seawater. The shrimps on day 8 after the feeding of kelp extracts were immersed in the virus-containing tank for 2 h (waterborne challenge). Then shrimps were returned to their original tank.

Survival was monitored until 10 days; the kelp extracts were administrated every day. In order to confirm the WSSV infection of the dead shrimps, RCR was done according to a previous report (Maeda et al., 1998). We have detected WSSV DNA from the lymphoid organs in all dead shrimps.

### Phenoloxidase Activity Measurement

A granulocyte suspension (600 μL of a 5 × 10^5^ cells/ml suspension) was placed in a 50 ml tube, and the cells were lysed using a sonicator (Kaijo Electric, Model 4280S vibrator, Japan). The cell lysate was transferred to a 1.5 ml tube and centrifuged (15,000 rpm, 5°C for 20 min), and 200 μL 0.1% trypsin (Difco) was then added to 200 μL supernatant and shaken using a plate mixer (Iuchi, TWIN MIXER TM-282, Japan) at 24°C for 30 min. Further, 200 μL 0.3% L-DOPA (Sigma) was added, and the mixture was shaken again at 24°C for 30 min using a plate mixer. Thereafter, the absorbance at 490 nm was measured using a spectrophotometer (JASCO, U-best30, Japan).

PO activity was determined using the following formula:

PO⁢Unit=⁢0.001⁢(absorbance⁢at⁢490⁢nm)/protein⁢amount⁢(mg)/reaction⁢time.

### Measurement of Superoxide Production

NBT (nitro blue tetrazolium, Sigma) stock solution consisted of 10 mg NBT dissolved in 1 ml distilled water and stored at 4°C protected from light. NBT working solution consisted of NBT stock solution diluted with culture buffer to 0.1%. As a stimulant, fMLP was adjusted to 1.3 μg/ml with culture buffer.

To measure superoxide production, separated granulocytes (100 μL of a 5 × 10^5^ cells/ml suspension) from each sample were placed into eight wells of a 96-well plate coated with Poly-L-lysine (Sigma), and incubated at 24°C for 30 min, after that the supernatant was removed. Subsequently 100 μL culture buffer was added in two wells, 50 μL culture buffer plus 50 μL SOD (Bovine liver-derived superoxide dismutase 300 units/ml in culture buffer) was added in two wells, 50 μL culture buffer and 50 μL fMLP was added in two wells, and the remaining two wells were filled with 50 μL fMLP and 50 μL SOD. Finally, 50 μL NBT working solution was added to all wells and incubated at 24°C for 2 h. After removing the supernatant, samples were fixed in 200 μL methanol for 5 min, washed twice with 70% methanol and air dried. Thereafter, 120 μL 2 mol/l KOH and 140 μL DMSO were added, and after vigorous agitation with a pipette, the absorbance at 630 nm was measured using a spectrophotometer.

Measured values were obtained by calculating the average value for each duplicate treatment described. To calculate superoxide production for each sample, the value of the SOD-containing wells was subtracted from the SOD-free wells, and multiplied by 1,000.

### Statistical Analysis

Statistical evaluation of differences between experimental groups was conducted using a Man-Whitney U test. To analyze survival rate, a Log-rank (Mantel-Cox) test was used. *P* < 0.05 was considered statistically significant.

## Results

### Setup of Boyden Chamber Chemotaxis Assay

In order to confirm the purity and function, the isolated hemocytes (granulocytes) were incubated with yeast. Granulocyte ware looked like same shape and staining pattern, and they phagocytosed the yeast (Figure 1A). This indicates that we could separate granulocyte population with function.

The first set of experiment, we compared the membrane and fixation method, to know the best condition for adherence of granulocytes to the membrane. PVP and PVPF were used as membrane filters, and the number of cells attached to the filter was measured using either 20% formalin or 1% glutaraldehyde for fixation (Figure 2A). The fixation method does not affect the number of cells present, but that the adherence rate of blood cells was significantly increased with PVP membrane relative to PVPF (*P* < 0.05).

The second set of the experiment, we examined the relationship between incubation and random migration (Figure 2B). Because the cells move randomly without any chemoattractant. After incubating for 2, 4, 6 h, the number of cells migrating to the lower surface of the filter was 1.1 ± 0.4, 2.9 ± 0.6, 2.7 ± 0.7, respectively, indicating random migration plateau at 4 h. The pH of culture medium affects many biological processes, so the third set of the experiment, we examined the pH of the culture buffer on random migration (Figure 2C). The lower chamber was adjusted to pH 7.0, 7.4, and 7.6, and 2.9 ± 0.6, 1.8 ± 0.4, 0.9 ± 0.4 cells, respectively migrating to the lower surface of the filter after 4 h. The highest random migration was seen in pH 7.0.

### Investigation of Chemotactic Factor

The fourth set of the experiment, we decide the optimal concentration of chemoattractant. fMLP, which is a formyl peptide derived from bacteria, is a well-known chemotactic factor (Lindemann et al., 2015) so was selected for this study and examined the optimal concentration (Figure 2D). Highest migration was seen in 10^– 8^ mol/L. The fifth set of the experiment, we tested the incubation time with fMLP (10^– 8^ mol/L) in Figure 2E. The number of migrating cells at 2, 4, and 6 h were 7.3 ± 2.8, 11.5 ± 2.8, and 11.1 ± 3.6, respectively. Four hours incubation reached to the plateau of migration.

The sixth set of the experiment, we tested the pH with fMLP (Figure 2F). The migrating cell numbers at pH 7.0, 7.4 and 7.6 were 11.5 ± 2.8, 6.0 ± 1.6, and 6.0 ± 2.3, respectively. The highest migration was seen in pH 7.0 so that optimal for the culture buffer is pH 7.0. The filter pore size is impact factor for this assay so that we conducted the seventh set of experiment (Figure 2G). The number of migrating cells at pore sizes 3, 5, and 8 μm were 2.3 ± 0.4, 11.5 ± 2.8, 5.6 ± 0.9, respectively, and it was revealed that the optimal filter pore diameter was 5 μm.

### Checkerboard Assay

In order to clarify whether chemotactic factors elicit migration of test cells, we conducted checkerboard assay for final set of experiment (Figures 2H,I). The cells migrated in an fMLP concentration gradient-dependent manner, and the results satisfied all of the criteria described in the section “Materials and Methods.”

### Kelp Extracts Enhanced Mj Hematocyte Chemotaxis

We then determined whether the chemotaxis assay was applicable for development of oral immunostimulants against WSSV.

As a candidate immunostimulant, we obtained Kelp extracts (HE and CL), which contained the β-glucan laminaran (Figure 3A). Laminaran is a β-1,3-glucan with one β-1,6-side chain for every four β-1,3-linked glucose residues (Ahmadi et al., 2015; Figure 3C). According to the yield rates illustrated in Figure 3B, we compared the efficiency of CL extraction starting from dried kelp or HE, and detected a similar efficiency at 2.9% vs. 3.1% (21.1 × 14.7% = 3.1%). Therefore, HE contained all of the CL and extra. Kelp extracts were mixed with normal feed, which was fed to Mj for 3–7 days prior to the chemotaxis assay (Figure 3D). There was no difference between day 0 and day 3 of feeding, but on day 7, HE and CL enhanced Mj hematocyte chemotaxis (Figure 3E).

### Kelp Extracts Conferred Protection Against WSSV

Enhanced hematocyte chemotaxis prompted us to determine whether the kelp extracts had a prophylactic effect against WSSV. On day 8 of feeding, Mj were infected with WSSV (Figure 3F). The HE and CL-administrated groups demonstrated improved survival rates relative to the control group (Figure 3G).

### Activation of Mj Hemocytes With Kelp Extracts

**FIGURE 4:**
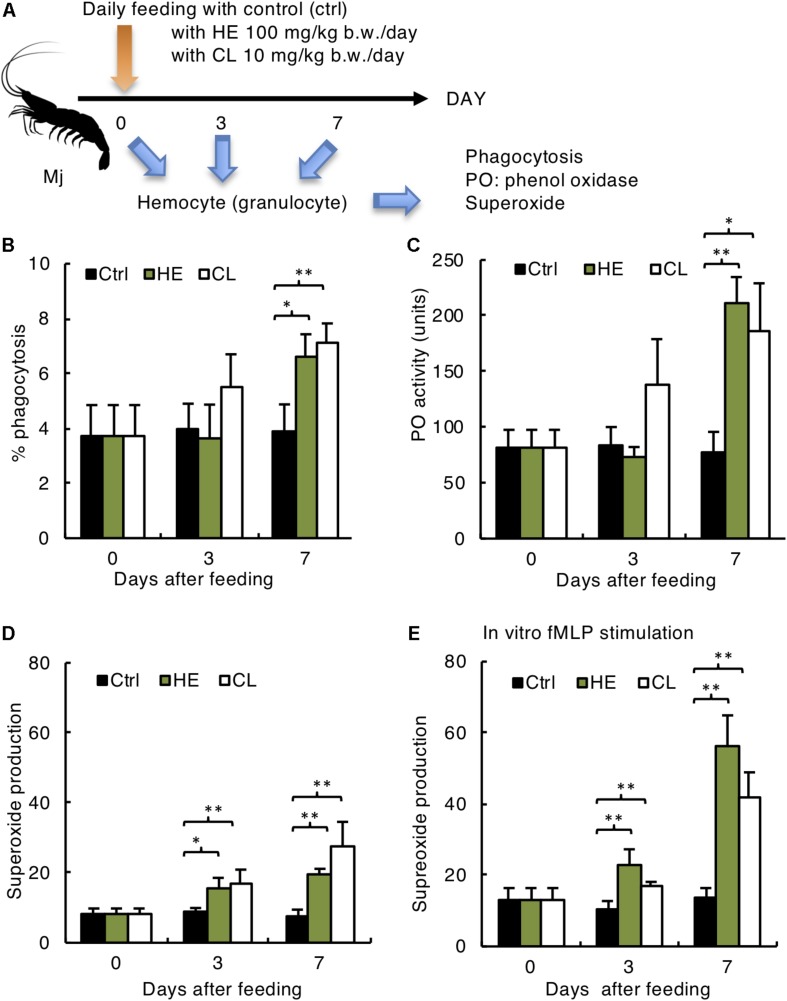
Activation of Mj host defense system by Kelp extracts. **(A)** Experimental design. **(B)** Phagocytosis rate of yeast, **(C)** phenol oxidase activity, **(D)** superoxide production without *in vitro* stimulation, and **(E)** superoxide production with *in vitro* fMLP stimulation of Mj granulocytes. **(B–E)** *N* = 6 in/group. ^∗^*P* < 0.05, ^∗∗^*P* < 0.01.

Finally, we determined if kelp extract enhancement of chemotaxis and protection against WSSV corresponded with activation of other immunological functions, including phagocytosis, PO activity and superoxide production (Figure 4A). Kelp extracts increased phagocytosis (Figure 4B) and PO activity (Figure 4C) on day 7 after administration. However, superoxide production was activated as early as day 3 after administration, with significant enhancements on day 3 in the both HE and CL groups (Figure 4D). Further, *in vitro* fMLP stimulation increased superoxide production, suggesting the assay’s validity and relevance (Figure 4E). Taken together, these results suggest that hematocyte chemotaxis is a good indicator of immunological function, and could be useful for development of oral immunostimulants in shrimp.

## Discussion

In this study, the optimum conditions of the Boyden chamber method for shrimp granulocyte chemotaxis were examined. fMLP acts as a chemotactic factor for hemocytes from Mj, and also for hemocytes from the hard clam *Mercenaria mercenaria* (Fawcett and Tripp, 1994). Our conclusion of the optimal conditions for chemotaxis of granulocytes from Mj with Boyden chamber are: 5 μm pore size Polyvinylpyrrolidone membrane; culture buffer at pH 7.0; and chemotactic factor formyl-methionyl-leucyl-phenylalanine 10^–8^ mol/L; 4 h incubation time.

The shrimp host defense is supported by humoral and cellular factors. PO activity is widely present in insects and crustaceans (Loker et al., 2004). PO is produced by a serine protease, in which blood cell proPO is specifically activated by β-1,3-glucan or peptidoglycan. PO then oxidizes substrates such as tyrosine and dopa to form melanin (Loker et al., 2004). Melanin, the final product of the proPO cascade reaction, is deposited in the lymphoid organs of shrimp infected with pathogens, including Vibrio disease and Fusarium, appearing to surround the sites of infection. This suggests that PO activity plays an important role in shrimp immunity.

One of the main host defenses perpetuated by hemocytes is phagocytosis of foreign bodies. Some molecules, such as β-1,3-glucan, peptidoglycan and lipopolysaccharide, also up-regulate phagocytosis. Oral administration of β-1,3-glucan derived from *Schizophyllum commune*, peptidoglycan derived from *Bifidobacterium thermophilum* and lipopolysaccharide derived from *Pantoea aggulomerans* increase phagocytic activity in shrimp and prawns, conferring a protective effect against bacterial and viral infections (Itami et al., 1998; Chang et al., 2000; Takahashi et al., 2000). In the present study, kelp extract containing the β-glucan laminaran increased the phagocytic activity of shrimp granulocytes.

A protein with a molecular weight of 36 kDa that recognizes β-1,3-glucan has been isolated from crayfish (*Pacifastacus leniusculus*) blood (Duvic and Soderhall, 1990). This suggests that the same type β-1,3-glucan (laminaran) receptor could potentially be present in shrimp blood cells, and that blood cell phagocytosis could be activated by laminaran binding to the receptor.

Shrimp blood cells are known to produce reactive oxygen species, similarly to mammalian neutrophils and macrophages (Song and Hsieh, 1994). Phagocytic cells first recognize and activate various chemotactic factors released from infected cells, and then migrate to the location of the opsonized bacteria. Phagocytic cells recognize and engulf foreign substances such as bacteria using receptors such as the Toll-like receptor (TLR). Phagocytosed bacteria are then exposed to various bactericidal mechanisms within the phagosome. The O_2_-dependent bactericidal mechanism is triggered by the reduction of oxygen by phagosomal membrane enzymes, and the subsequent production of a superoxide anion as a bactericidal ROS. In addition, various other ROSs (hydroxy free radicals, singlet oxygen, hydrogen peroxide) are produced. However, the bactericidal effect of this pathway does not require fusion with lysosomes, and is automatically activated by phagosome formation (Dupre-Crochet et al., 2013). ROSs are harmful to normal tissues, but are powerful weapons against non-self cells.

When vertebrate neutrophils are exposed to bacteria or chemotactic factors, intracellular NADPH increases, and cell membrane NADPH oxidase is activated and exposed to the cell membrane surface, releasing ROS. It is thought that this ROS reacts with iron ions to produce more ROS, which confers bactericidal action by modifying bacterial cell membranes and intracellular enzymes (Laroux et al., 2005). Similarly, shrimp blood cells are also known to produce ROS to confer bactericidal activity (Song and Hsieh, 1994; Muñoz et al., 2000).

In a report by Chang et al. (2000), β-1,3-glucan derived from *Schizophyllum commune* was orally administered to *Penaeus monodon*, which increased ROS production capacity. In the present study, we identified a similar effect of kelp extract. Furthermore, when hemocytes were stimulated with fMLP, superoxide production increased significantly. Therefore, we deduced that when pathogens such as bacteria or viruses enter the living shrimp fed kelp extract, bacterial β-glucan, peptidoglycan and LPS further enhance the ability to produce superoxide and kill invading organisms, protecting against infection.

In the present study, we optimized the Boyden chamber chemotaxis assay, and established its utility in development of oral immunostimulants for shrimp in aquaculture.

## Data Availability Statement

All datasets generated for this study are included in the article/supplementary material.

## Author Contributions

TI performed the experiments, analyzed the results, created the figures, and wrote the manuscript. YT supervised the project. Both authors contributed to the discussion of the results and preparation of the manuscript.

## Conflict of Interest

The authors declare that the research was conducted in the absence of any commercial or financial relationships that could be construed as a potential conflict of interest.
